# Polygenic scores for schizophrenia and general cognitive ability: associations with six cognitive domains, premorbid intelligence, and cognitive composite score in individuals with a psychotic disorder and in healthy controls

**DOI:** 10.1038/s41398-020-01094-9

**Published:** 2020-11-30

**Authors:** Magnus Johan Engen, Siv Hege Lyngstad, Torill Ueland, Carmen Elisabeth Simonsen, Anja Vaskinn, Olav Smeland, Francesco Bettella, Trine Vik Lagerberg, Srdjan Djurovic, Ole A. Andreassen, Ingrid Melle

**Affiliations:** 1 NORMENT, Division of Mental Health and Addiction, Oslo University Hospital & Institute of Clinical Medicine, University of Oslo, Oslo, Norway; 2 Department of Psychology, Faculty of Social Sciences, University of Oslo, Oslo, Norway; 3 Early Intervention in Psychosis Advisory Unit for South East Norway, Division of Mental Health and Addiction, Oslo University Hospital, Oslo, Norway; 4 NORMENT, Department of Clinical Science, University of Bergen, Bergen, Norway; 5 Department of Medical Genetics, Oslo University Hospital, Oslo, Norway

**Keywords:** Clinical genetics, Predictive markers

## Abstract

Cognitive impairments are considered core features in schizophrenia and other psychotic disorders. Cognitive impairments are, to a lesser degree, also documented in healthy first-degree relatives. Although recent studies have shown (negative) genetic correlations between schizophrenia and general cognitive ability, the association between polygenic risk for schizophrenia and individual cognitive phenotypes remains unclear. We here investigated the association between a polygenic score for schizophrenia (SCZ_PGS_) and six well-defined cognitive domains, in addition to a composite measure of cognitive ability and a measure of premorbid intellectual ability in 731 participants with a psychotic disorder and 851 healthy controls. We also investigated the association between a PGS for general cognitive ability (COG_PGS_) and the same cognitive domains in the same sample. We found no significant associations between the SCZ_PGS_ and any cognitive phenotypes, in either patients with a psychotic disorder or healthy controls. For COG_PGS_ we observed stronger associations with cognitive phenotypes in healthy controls than in participants with psychotic disorders. In healthy controls, the association between COG_PGS_ (at the *p* value threshold of ≥0.01) and working memory remained significant after Bonferroni correction (*β* = 0.12, *p* = 8.6 × 10^−5^). Altogether, the lack of associations between SCZ_PGS_ and COG_PGS_ with cognitive performance in participants with psychotic disorders suggests that either environmental factors or unassessed genetic factors play a role in the development of cognitive impairments in psychotic disorders. Working memory should be further studied as an important cognitive phenotype.

## Introduction

Cognitive impairment is a core feature of schizophrenia and other psychotic disorders^1^. Impairments are most prominent in schizophrenia^2–5^, but also frequent in bipolar disorders^3,6–8^. The genetic contribution to the risk of developing a psychotic disorder is high, with heritability estimates for schizophrenia and bipolar disorder ranging from 0.6 to 0.8^9^. Both disorders show high degrees of polygenicity and have a considerable genetic overlap^10^. The genetic contribution to cognitive abilities is also substantial with a heritability estimate of 0.5^11^. It is now known that the phenotypic link between cognitive domains and a number of different psychiatric diagnoses is at least partly explained common genetic variants^12^. Mild cognitive impairments have been found in first-degree relatives of probands with both schizophrenia and bipolar disorder^13,14^, and a recent study estimated that as much as one-third of the genetic risk for schizophrenia could be mediated through cognition-relevant pathways^15^. Understanding the genetic underpinnings of cognitive phenotypes in psychotic disorders could be key in understanding the etiology of these disorders. The considerable negative impact of cognitive impairment on functional outcome and our current lack of efficient treatment strategies emphasize the importance of identifying the underlying mechanisms^16,17^.

The wealth of data uncovered by large-scale genome-wide association studies (GWAS) offers promising research opportunities for exploring the genetic architecture of psychotic disorders. The Psychiatric Genomics Consortium (PGC) identified 108 separate genetic loci statistically associated with schizophrenia risk after correction for multiple testing, which accounted for 3.4% of the variation in liability to schizophrenia^18^. These loci meet the high standard set for “true-positive” hits, but applying more liberal *p* value thresholds (PTs) reveals that the actual polygenic architecture of schizophrenia comprises thousands of genetic variants, each with very small effects that in aggregate explain a third to a half of the heritability of the disorder^18^. This is the basis for the calculation of polygenic scores (PGSs). Here common allele associations from a GWAS discovery sample are used to calculate an aggregate PGS as the cumulative effect sizes that differ between cases and controls. PGSs can be created for any investigated phenotype and can be used to explore the associations between the polygenic basis of this phenotype and other individual characteristics.

How the PGS for schizophrenia (PGS_SCZ_) is related to cognition has been studied in both clinical samples and in the general population. Results from studies of clinical samples have been mixed: While one study found strong negative associations between PGS_SCZ_ and seven cognitive domains^19^, other studies have reported a lack of significant associations^20–23^. Two studies have in addition found PGS_SCZ_ to be negatively associated with general cognitive ability in healthy controls but not in patients^24,25^. A plausible explanation for the latter findings is that the effect of the association between PGS_SCZ_ and cognition is overshadowed by non-genetic illness factors in patients, creating stronger associations between genetic and cognitive measures in the healthy control group than in the patient group. In line with this, one of these studies^25^ found that higher PGS for intelligence was associated with better general cognitive ability in both patients and healthy controls, but with a stronger effect in healthy controls. However, the study did not explore the effect on specific cognitive domains.

As there is no consensus on the operationalization of cognitive domains, studies vary in their selection of cognitive tests. In genetic studies, general cognitive ability is frequently operationalized as a composite score of available tests referred to as Spearman’s *g*, a term for the latent variable thought to underlie all cognitive domains^26^. Because *g* is confirmed as a valid construct^26,27^ with high heritability^26,28^, and allows for the combination of unrelated cognitive tests to increase sample size and statistical power, its frequent use in genetic studies is unsurprising. However, substantial and independent genetic effects on specific cognitive domains have also been demonstrated^27^. The variation in both sample sizes and cognitive phenotypes used in studies of PGS_SCZ_ makes interpretation of results challenging^29^, and at present only one study has investigated how the PGS_SCZ_ is associated with a broader selection of cognitive phenotypes known to be influenced by psychosis in both individuals with psychosis and healthy controls^19^.

In summary: While a genetic basis for cognitive impairment in psychotic disorders appears established^15,30,31^, the nature of this association is complex and its true extent remains unclear. Twin studies indicate that the shared genetic basis for schizophrenia and cognition is substantial^32,33^. However, a population-based twin study with measures of premorbid cognitive ability found only 7% shared genetic variance between psychosis and cognition^34^. In addition, while the association between *g* and cognitive domains are considerable, they are not identical and probably have different genetic architectures with different associations to the genetic basis of psychotic disorders^35^.

To increase our understanding of the genetic relationship between psychotic disorders and cognitive function, we here investigated the association between PGS_SCZ_ and a well-defined set of cognitive domains in a large sample of participants with a psychotic disorder and healthy controls. In addition, we explored the associations between the PGSs for general cognition (PGS_COG_) based on a GWAS with stringent inclusion criteria, and the same set of cognitive domains in the same group of participants. Our hypothesis was that we would find negative associations between the PGS_SCZ_ and cognitive domain scores and positive associations between and PGS_COG_ and cognitive domain scores in both groups, but that the effect size of the associations would be higher in healthy controls. We also included an estimate of premorbid intelligence, and hypothesized less differences between participants with psychosis and healthy controls for this measure.

## Methods

### Participants, clinical measures, and study design

The current study was based on the inclusion criteria for the thematically organized psychosis study. All recruited participants were between 18 and 65 years. For the research questions in the current study, we included participants with declared European ancestry who had participated in cognitive assessment and met the criteria for a psychotic disorder. Psychotic bipolar disorder was included based on genetic overlap with schizophrenia and the phenotypic similarity with regards to cognitive impairment. The sample included participants with schizophrenia spectrum disorders (*N* = 522) (schizophrenia *n* = 294, schizophreniform disorder *n* = 28, schizoaffective disorder *n* = 72, and psychosis not otherwise specified *n* = 128) and psychotic bipolar spectrum disorders (*n* = 209) (bipolar I disorder *n* = 170, bipolar II disorder *n* = 28, bipolar disorder not otherwise specified *n* = 11), resulting in a clinical sample of 731 psychosis spectrum participants. Clinical interviews for DSM-IV (Diagnostic and Statistical Manual of Mental Disorders, fourth edition) diagnoses in patients^36^ were performed by psychologists, psychiatrists, or medical doctors. Healthy controls (*n* = 851) were randomly selected from the population registry in the same catchment area. The healthy controls were screened with the Primary Care Evaluation of Mental Disorders (PRIME-MD)^37^ and a family history interview, performed by individuals with a master’s degree in psychology or neuroscience. Controls were not included if they had a personal or family (first-degree) history of a psychotic disorder. Both psychosis spectrum participants and healthy controls were excluded if they did not speak a Scandinavian language, had a history of serious head injury, suffered from a known medical or neurological condition interfering with brain functioning, or intelligence quotient < 70. Demographic and clinical characteristics are presented in Table 1.

**Table 1:** Demographic and clinical variables.

	Healthy controls	Individuals with psychosis
*N* Total (% females)	851 (47)	731 (48)
Mean age (SD)	32.7 (9.0)	31.3 (10.8)
Education years (SD)	14.4 (2.2)	12.8 (2.6)
AP medication^a^ (SD)		1.0 (0.7)
Duration of illness^b^ (SD)		6.3 (7.4)

*SD* standard deviation, *AP* antipsychotic.

^a^Daily antipsychotic dosage relative to the average recommended daily dosage. One hundred and seventy-five individuals with missing data.

^b^Years from first episode with psychosis or mania. Twenty-seven individuals with missing data.

The Positive and Negative Syndrome Scale^38^ Wallwork’s five factor model^39^ was used to measure symptoms. The Alcohol Use Disorder Identification Test and the Drug Use Disorder Identification Test^40^ were used to assess alcohol use and drug use, respectively. Duration of illness was based on information from the severe combined immunodeficiency interview, and was calculated as the time in years from the first episode of psychosis or mania. Use of antipsychotic medication was recoded into defined daily dosages^41^.

All participants in the cognitive assessments were included to increase statistical power. However, some participants were lacking scores for one or more subtests, precluding the calculation of a cognitive composite score for these individuals. Therefore, the sample varies from *n* = 726 for verbal learning to *n* = 533 for the cognitive composite score. Mean scores and *N*s for participants with psychosis and healthy controls for all tests and domains are presented in Table 2. Written informed consent was obtained from all participants.

**Table 2:** Cognitive assessment scores.

	Psychosis		Controls	
	*N*	Mean ± SD	*N*	Mean ± SD
Cognitive domain				
** Verbal learning**	**726**	**−0.75** **±** **1.27**	**850**	**0.00** **±** **1.00**
CVLT-II verbal learning^a^	537	50.18 ± 11.79	380	57.14 ± 9.73
HVLT verbal learning^b^	189	24.87 ± 5.58	472	28.23 ± 3.94
** Verbal memory**	**685**	**−0.69** **±** **1.31**	**850**	**0.00** **±** **1.00**
CVLT-II delayed free recall^a^	535	11.42 ± 3.37	380	13.21 ± 2.69
HVLT delayed free recall^b^	150	8.85 ± 2.60	472	10.20 ± 1.73
**Processing speed**	**679**	**−0.96** **±** **1.13**	**845**	**0.00** **±** **0.78**
WAIS digit-symbol coding^a^	536	62.03 ± 15.56	380	77.46 ± 13.23
BACS digit-symbol coding^b^	189	47.43 ± 10.90	472	58.31 ± 9.15
D-KEFS color naming*^d^	682	33.23 ± 7.16	847	28.16 ± 4.64
D-KEFS word reading*^d^	682	23.56 ± 5.02	846	21.25 ± 3.71
Executive functions				
**Working memory**	**564**	**−0.60** **±** **0.88**	**812**	**0.00** **±** **0.88**
WAIS letter-number sequencing^a^	461	9.47 ± 2.38	366	11.51 ± 2.56
MCCB letter-number sequencing^b^	184	13.58 ± 3.17	472	15.17 ± 3.03
WAIS digit span^d^	642	14.74 ± 3.64	827	16.44 ± 3.71
**Attentional control**	**678**	**−0.92** **±** **1.47**	**824**	**0.00** **±** **0.86**
D-KEFS inhibition*^,d^	682	59.00 ± 16.51	845	48.55 ± 9.79
D-KEFS inhibition-switching*^,d^	678	64.26 ± 17.76	825	55.13 ± 11.67
**Category fluency**	**693**	**−1.06** **±** **1.20**	**850**	**0.00** **±** **0.99**
D-KEFS category fluency^a^	505	40.58 ± 9.65	381	49.25 ± 7.85
MCCB category fluency^b^	188	23.03 ± 6.44	470	28.46 ± 5.87
**General cognitive ability**				
**NART**^c,d^	**635**	**−0.47** **±** **1.19**	**825**	**0.00** **±** **1.00**
**Cognitive composite score**	**533**	**−0.81** **±** **0.89**	**802**	**0.00** **±** **0.61**

All domains in bold are transformed into *z*-scores using the healthy control mean and standard deviation. The cognitive composite score is averaged over all domains except NART, which is an estimate of premorbid intellectual functioning.

*Scores reversed when transformed into *z*-scores to make higher values reflect better performance.

^a^Test from Battery 1.

^b^Test from Battery 2.

^c^Test included in both assessment batteries.

### Genetic analyses

DNA samples obtained from blood or saliva were sent for genotyping in six separate batches between the years of 2014 and 2017 at deCODE Genetics (Reykjavik, Iceland). The samples were analyzed using Illumina Human OmniExpress12 (first four batches), Infinium OmniExpress24 (fifth batch), and Illumina Global Screening Array (sixth batch) platforms, respectively. Identical but independent procedures were followed for sample batches genotyped on different platforms. PLINK version 1.9^42^ was deployed to perform the necessary pre-imputation quality control. This involved removal of any samples with possible contamination (heterozygosity more than five standard deviations above the mean) or too low coverage ((<80%) to enable its detection) and any variants with genotyping rate lower than 95%, Hardy–Weinberg disequilibrium test *p* value lower than 10^−4^, and high rate of Mendel errors (https://www.cog-genomics.org/plink/1.9/basic_stats#Mendel) in eventual trios or significant (false discovery rate < 0.5) batch effects (in case multiple batches were being processed simultaneously). The quality-controlled genotypes were phased using Eagle^43^, and missing variants were imputed with MaCH^44,45^ using version 1.1 of the trans-ethnic reference sample put together by the haplotype reference consortium (HRC)^46^. High-quality variant sets from the quality control procedure (see Supplementary information) were selected to impute each individual’s sex and compute each individual’s genetic principal components (PCs). The latter consisted of the individual’s components along the 20 first eigenvectors of the pairwise genetic covariance matrix of a sub-sample of unrelated individuals from the reference (HRC) panel. Following the quality control and imputation procedure, variants with information score <0.8 or minor allele frequency <0.01 were removed. In addition, individual genotypes imputed with <75% confidence were set to missing, and the remaining ones were converted to best guess hard allelic dosages. Finally, individuals with imputation rate <95% and an up to third-degree relative with better imputation rate or differing annotated and imputed sex were excluded. For more detailed descriptions of the quality control and imputation procedures, refer to the Supplementary information.

### Polygenic scores

The SCZ_PGS_ were computed following the method described by Purcell et al.^47^. A meta-analysis covering all PGC sub-studies, except our sample, was performed to obtain risk allele effect sizes [ln(OR)] for all imputed variants. We then used PRSice^48^ to compute PGSs based on seven increasing PTs: PT ≤ 5 × 10^−8^, PT ≤ 1 × 10^−5^, 0.001, 0.01, 0.05, 0.1, and 0.5. The SCZ discovery sample used for SCZ_PGS_ is based on 36,989 participants with schizophrenia and 113,079 healthy controls. Participants come from a total of 49 case–control samples (46 European and 3 East Asian) and 3 family-based samples of European ancestry^18^.

The PGS_COG_ were computed following the same procedure for seven increasing PTs: PT ≤ 5 ×10^−8^, PT ≤ 1 × 10^−5^, 0.001, 0.01, 0.05, 0.1, and 0.5, but using a meta-analysis of COGENT consortium sub-studies of general cognitive function^49^. This discovery sample consists of 35,298 individuals with European ancestry from 24 studies (mean age of 45.6 (SD ± 8.6) 51.4% females). Most of these studies are of clinical populations (schizophrenia, Alzheimer’s disease, cardiovascular disorders), but the healthy controls and participants in studies of the general population were also included.

### Measures of cognition

All participants were assessed with a comprehensive cognitive assessment battery. At the start of the study, there was no international consensus regarding which tests to use and the study constructed a battery based on the knowledge about cognitive domains that were most affected by psychotic disorders (“battery 1”). With the development of the Measurement and Treatment Research to Improve Cognition in Schizophrenia (MATRICS) Consensus Cognitive Battery (MCCB)^50^, the study decided to change to the MCCB (“battery 2”). Since both batteries focused on domains affected by psychotic disorders, they covered essentially the same areas, but used somewhat different specific tests. For the purpose of the study results from the two batteries were merged according to the following procedure^51^: Cognitive test scores were first converted into *z*-scores based on the mean scores and standard SDs of the healthy control group. Tests were combined into the six specific cognitive domains based on their well-recognized relevance to cognitive impairment in psychotic disorders^52^, and included Attentional control, Category fluency, and Working memory (these three covering different aspects of Executive function) together with Processing speed, Verbal learning, and Verbal memory.

*Attentional control* comprised the inhibition and the inhibition/switching conditions of the Delis-Kaplan Executive Functions System (D-KEFS) Color-Word Interference task^53^; *Category fluency* was constructed merging the Category fluency test from D-KEFS^53^ and the Category fluency test from the MCCB^50^.

*Processing speed* was measured using the word reading and color naming conditions of the Color-Word Interference task from the D-KEFS^53^. In addition, the digit symbol tests from WAIS-III^54^ and BACS^50,55^ were included. *Working memory* consisted of the WAIS-III digit span, forward and backward, and the WAIS-III Letter-Number Sequencing test, merged with Letter-Number Span from the MCCB^50^. The *Verbal learning* and *Verbal memor*y domains were constructed merging relevant conditions from the California Verbal Learning Test II and the Hopkins Verbal Learning Test-Revised, respectively^50,56^.

In addition to investigating these six domains separately, we also combined them into a *Cognitive composite* score as a measure of general cognitive ability. The cognitive composite score was constructed as the individual’s mean *z*-score across the six domains. Finally, the National Adult Reading Test (NART)^57^ was included as an estimate of premorbid intelligence. The Norwegian NART is a valid measure of premorbid intelligence^58^.

### Statistical analyses

**Fig. 1:**
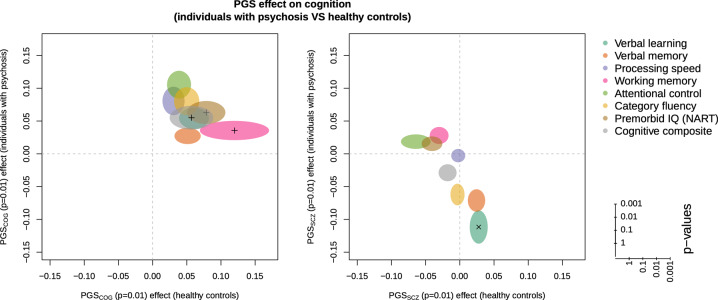
The left and right figures present PGS_COG_ and PGS_SCZ_ associations with the eight cognitive phenotypes at *P*_T_ level = 0.01, respectively. The axes denote the associations (*β*) to healthy controls (*x*) and individuals with psychosis (*y*). The size and shape of the bubbles on both plots represent the significance of the association.

We used SPSS version 25 for the analyses of the main research question. Means are reported for data with normal distribution and all test are two-tailed with *α* set to *p* < 0.05. We started the analyses by selecting the relevant ancestry PCs to be included in the analyses, by bivariate association analyses between the 20 first ancestry PCs, and the ten PGS_SCZ_ *P*_T_ levels and case–control status, resulting in a list of 16 PCs that were associated at the *p* ≤ 0.05 level with any of these. The next step was to run ten separate logistic regressions (one for each *P*_T_) with case–control status as the dependent variable correcting for genotyping batch and the 16 PCs to identify the *P*_T_ with the highest explained variance in case–control status. The *P*_T_ with the best fit based on the Akaike information criterion was *P*_T_ ≤ 0.01 (Fig. 1). *P*_T_ ≤ 0.01 was thus selected for testing for associations between PGS_SCZ_ and the cognitive phenotypes in the subsequent analyses. For the PGS_COG_, all available *P*_T_ levels were used in the main analyses.

The main analyses to investigate the association between PGS_SCZ_ *P*_T_ ≤ 0.01 and cognitive domains were multiple linear regression analyses, with the different cognitive phenotypes as dependent variables and the PGS_SCZ_ as the independent variable introduced at the last step of the analysis after, controlling for age, sex, genotyping batch, and PCs. For this step, we selected PCs that had a bivariate association at the *p* ≤ 0.05 level to either the PGS_SCZ_ *P*_T_ ≤ 0.01, the seven PGS_COG_ *P*_T_ levels or to any of the eight cognitive phenotypes, resulting in nine PCs. For the next research question, the multiple linear regression analyses were repeated, this time with the seven PGS_COG_ *P*_T_ levels as independents. All these analyses were done separately for psychosis participants and for healthy controls. The *α* level was set at 0.05 for all tests, with Bonferroni corrections. We adjusted for testing in two different populations for eight domains and one PGS_SCZ_ *P*_T_, yielding a corrected threshold of *p* < 0.003 (16 tests) for PGS_SCZ_ and likewise yielding a corrected threshold of *p* < 0.00045 (112 tests) for the seven PGS_COG_ *P*_T_ levels. The difference in regression coefficients between cases and controls were tested using the method of Weaver and Wuensch, taking the sample size, the number of independent variables, and the standard error into account^59^. In the follow-up analyses, all regressions were repeated with only participants within the schizophrenia spectrum.

## Results

### The PGS_SCZ_ predicted case–control status but was not significantly associated with any cognitive domain

The associations between the PGS_SCZ_ at *P*_T_ ≤ 0.01 and the cognitive phenotypes are displayed in Fig. 1. Except for a nominally significant association to verbal learning for individuals with psychosis (*β* = −0.11, *p* = 0.03), there were no significant associations between the PGS_SCZ_ and the remaining cognitive phenotypes (numerical values for *β*s, 95% confidence intervals (CIs), and *p* values for both participants with psychosis and healthy controls are presented in Supplementary Table S1). Repeating the analyses, including only participants within the schizophrenia spectrum, did not change the results.

### The PGS_COG_ predicted working memory performance in controls

The analyses were repeated using the seven pre-selected PGS_COG_ *P*_T_ levels to evaluate how the genetic basis for general cognition was associated with the cognitive phenotypes. There were no associations between PGS_COG_ and cognitive phenotypes that reached unadjusted levels of statistical significance across the seven *P*_T_s in participants with a psychotic disorder. In healthy controls, we observed the strongest associations between PGS_COG_ and cognitive phenotypes at *P*_T_ ≤ 0.01, where nominally significant results were for the cognitive composite score (*β* = 0.06, *p* = 0.01), NART (*β* = 0.08, *p* = 0.02), and working memory (*β* = 0.12, *p* = 0.00009). Only the association with working memory reached the Bonferroni-corrected level of significance. The adjusted *R*^2^ for this model was 0.022 and the change in adjusted *R*_2_ when entering the PGS_COG_ at the last step was 0.017. The association between cognitive phenotypes and PGS_COG_ at *P*_T_ ≤ 0.01 are displayed for both psychosis participants and healthy controls in Fig. 1. (The numerical values for *β*s, 95% CIs, and *p* values for both psychosis participants and healthy controls at all *P*_T_s are presented as Supplementary material (Supplementary Table S2), while Supplementary Fig. S1 graphically shows the association at the six remaining *P*_T_ levels analyzed). Repeating the analyses including only patients within the schizophrenia spectrum did not change the results. The nominal *p* value for the difference in regression coefficients between patients and controls for the analysis of working memory and PGS_COG_ at *P*_T_ ≤ 0.01 was 0.05, below the level of statistical significance after correction for multiple testing.

### Symptoms and substance use did not moderate the association between PGS_COG_ and cognitive phenotypes in the psychosis sample

The analysis that yielded the strongest associations between the PGS_COG_ and cognitive phenotypes in healthy controls at *P*_T_ ≤ 0.01 (working memory) were repeated in participants with a psychotic disorder, controlling for clinical characteristics that potentially could influence the associations (illness history, symptom and substance use scores, and antipsychotic medication use). Clinical variables with associations *p* < 0.1 to the cognitive phenotypes (Supplementary Table S3) were entered in the analysis. This did not significantly alter the associations between PGS_COG_ and the cognitive phenotypes in the psychosis group. (The analysis for working memory and PGS_COG_ at *P*_T_ ≤ 0.01 is shown in Supplementary Table S4.) Repeating the analyses including only patients within the schizophrenia spectrum did not affect the results.

## Discussion

The current study is the largest to date investigating the association between a broad and well-defined set of cognitive domains and PGS_SCZ_ and PGS_COG_, in both healthy controls and participants with psychotic disorders. Overall, we did not find the hypothesized negative associations between PGS_SCZ_ and cognitive phenotypes. However, we did observe a statistically (Bonferroni-corrected) significant association between PGS_COG_ at *P*_T_ ≤ 0.01and working memory in healthy controls. This association in healthy controls was nominally stronger than in participants with psychotic disorders (*p* = 0.05).

A previous study, in a slightly smaller sample than the current, investigated the association between PGS_SCZ_ and estimates of premorbid and current cognitive abilities in patients with psychosis and healthy controls^24^. They found that premorbid intelligence was not associated with PGS_SCZ_, whereas better general cognitive ability was associated with lower PGS_SCZ_ in healthy controls. The negative finding for premorbid cognitive abilities was replicated in a large population-based study. The authors interpreted this to suggest that tests used to measure current cognitive abilities are more directly linked to the genetic risk factors of schizophrenia. In the present study, we also found that both measures of premorbid (NART) and current (composite score) cognitive abilities were not associated with PGS_SCZ_ at all (and only nominally associated with the PGS_COG_ in healthy controls). While some findings could be based on low statistical power, our study supports the notion that cognitive phenotypes differ in their associations with the genetic basis of schizophrenia.

Several previous studies have, however, found significant associations between PGS_SCZ_ and cognitive phenotypes, while others have not^20,22,23,60^. In a recent review of PGS_SCZ_ and cognitive functioning, the authors hypothesize that studies using improved cognitive phenotypes, such as the MCCB, will detect associations^29^. In line with this, a recent study including 127 patients and 136 healthy controls found significant associations between PGS_SCZ_ and all domains of the MCCB, including the cognitive composite score^19^. The present study investigated several tests from the MCCB in a significantly larger sample. This suggests that refining the cognitive phenotypes alone will not increase the power to detect associations. Since our sample size is larger than most of the previous studies reporting significant associations, our lack of findings is also not primarily based on lack of statistical power relative to these studies.

Recent studies indicate a complex pattern of association between the genetic basis for cognition and schizophrenia: While most (but not all) schizophrenia risk alleles were associated with lower intelligence, around half of bipolar disorder risk alleles were associated with higher intelligence^61^. Further, among the 75 loci *shared* between bipolar disorder and intelligence, there was an equal distribution of agonistic and antagonistic effect directions possibly explaining the overall non-significant correlation between these two phenotypes^61^. Another GWAS analysis reports a similar complex genetic relationship between schizophrenia and cognitive measures^35^. In line with this, other studies have found weak—and in some cases positive—associations between educational attainment as a proxy for cognitive abilities and schizophrenia^62^, despite behavioral data showing a strong negative association between schizophrenia and attaining higher education. Another study suggests that this may be caused by a positive association between educational attainment and risk alleles shared between schizophrenia and bipolar disorder, suppressing the negative association with risk alleles unique to schizophrenia^63^. Taken together, the findings indicate a highly complex and not uniformly negative association between schizophrenia risk variants and cognitive functioning that may explain the lack of association between PGS_SCZ_ and cognitive abilities.

Underpowered GWAS discovery samples or imprecision in current methods used to generate PGS_SCZ_ could account for lack of clear answers. The construction of the PGS_SCZ_ is powered by design to explain variance in case–control status and the phenotypic variance explained by the currently available PGS_SCZ_ is relatively low^64^. Here, increased sample sizes in the GWAS on which the PGS is based may generate PGSs with better predictive power and as the statistical power of GWASs increase, the improved PGS is may become a better tool for future personalized medicine^65^. As of now, any associations (or lack thereof) between PGSs and complex phenotypes should be interpreted with caution.

The finding regarding working memory is noteworthy. Working memory may be a particularly important domain because it has been suggested that it is at the core of the widespread deficits observed across psychotic disorders through its influence of proactive control^66^. According to theory, the inability to retain and organize information in short-term memory impairs the ability to proactively guide thoughts and behavior in an efficient manner and leads to observed impairments in several domains. A recent study of patients with psychotic disorders found significant associations between PGS for educational attainment and measures of working memory^60^; however, with a relatively limited influence on the explained variance. The diagnostic composition, sample size, and cognitive measures were approximately the same as the current. Educational attainment is perceived as a less precise proxy or indication of cognitive abilities; the larger sample size of educational attainment GWASs could, however, lead to a better calculation of the educational attainment PGS. The study does not report directly on findings in their somewhat smaller healthy control sample. Taken together, our results support the notion that clinical and environmental factors in persons with a psychotic disorder could explain more variance in cognitive phenotypes than in healthy controls, and that current estimates may overstate the role of cognition as a part of the genetic etiology of psychotic disorders. Future studies should include a focus on working memory.

## Supplementary information

Supplementary information
