# Supplementary material for: Polygenic scores for schizophrenia and general cognitive ability: associations with six cognitive domains, premorbid intelligence, and cognitive composite score in individuals with a psychotic disorder and in healthy controls
Source: Transl Psychiatry. 2020 Nov 30;10:416. doi: 10.1038/s41398-020-01094-9 (PMC7705731; doi:10.1038/s41398-020-01094-9)
Supplement: Supplementary file 1 — Supplementary information [file 41398_2020_1094_MOESM1_ESM.docx]

| **Attentional Control** |  |  |  |  |  |  |
| --- | --- | --- | --- | --- | --- | --- |
| **Healthy controls**  **Table S1.** Schizophrenia polygenic scores (PT ≤ 0.01) and associations to cognitive domains in healthy controls and individuals with psychosis (Psychosis). | **Estimate (Beta)** | **Standard error** | **T value** | ***p-*value** | **95% CI**  **lower bound** | **95% CI**  **upper bound** |
| PGS_SCZ_ P_T_ ≤ 0.01 | -0.064 | 0.035 | -1.835 | 0.066787 | -0.133 | 0.004 |
| **Psychosis** |  |  |  |  |  |  |
| PGS_SCZ_ P_T_ ≤ 0.01 | 0.019 | 0.067 | 0.278 | 0.781293 | -0.113 | 0.150 |
| **Category Fluency** |  |  |  |  |  |  |
| **Healthy controls** | **Estimate (Beta)** | **Standard error** | **T value** | ***p-*value** | **95% CI**  **lower bound** | **95% CI**  **upper bound** |
| PGS_SCZ_ P_T_ ≤ 0.01 | -0.003 | 0.041 | -0.084 | 0.933066 | -0.084 | 0.077 |
| **Psychosis** |  |  |  |  |  |  |
| PGS_SCZ_ P_T_ ≤ 0.01 | -0.062 | 0.051 | -1.218 | 0.223755 | -0.162 | 0.038 |
| **Composite score** |  |  |  |  |  |  |
| **Healthy controls** | **Estimate (Beta)** | **Standard error** | **T value** | ***p-*value** | **95% CI**  **lower bound** | **95% CI**  **upper bound** |
| PGS_SCZ_ P_T_ ≤ 0.01 | -0.018 | 0.026 | -0.699 | 0.484866 | -0.068 | 0.032 |
| **Psychosis** |  |  |  |  |  |  |
| PGS_SCZ_ P_T_ ≤ 0.01 | -0.029 | 0.043 | -0.670 | 0.503083 | -0.114 | 0.056 |
| **Premorbid IQ NART** |  |  |  |  |  |  |
| **Healthy controls** | **Estimate (Beta)** | **Standard error** | **T value** | ***p-*value** | **95% CI**  **lower bound** | **95% CI**  **upper bound** |
| PGS_SCZ_ P_T_ ≤ 0.01 | -0.041 | 0.040 | -1.023 | 0.306547 | -0.119 | 0.037 |
| **Psychosis** |  |  |  |  |  |  |
| PGS_SCZ_ P_T_ ≤ 0.01 | 0.015 | 0.052 | 0.296 | 0.767685 | -0.087 | 0.118 |
| **Precessing speed** |  |  |  |  |  |  |
| **Healthy controls** | **Estimate (Beta)** | **Standard error** | **T value** | ***p-*value** | **95% CI**  **lower bound** | **95% CI**  **upper bound** |
| PGS_SCZ_ P_T_ ≤ 0.01 | -0.002 | 0.031 | -0.076 | 0.939215 | -0.064 | 0.059 |
| **Psychosis** |  |  |  |  |  |  |
| PGS_SCZ_ P_T_ ≤ 0.01 | -0.003 | 0.050 | -0.056 | 0.955343 | -0.100 | 0.095 |
| **Verbal Learning** |  |  |  |  |  |  |
| **Healthy controls** | **Estimate (Beta)** | **Standard error** | **T value** | ***p-*value** | **95% CI**  **lower bound** | **95% CI**  **upper bound** |
| PGS_SCZ_ P_T_ ≤ 0.01 | 0.028 | 0.039 | 0.701 | 0.483774 | -0.050 | 0.105 |
| **Psychosis** |  |  |  |  |  |  |
| PGS_SCZ_ P_T_ ≤ 0.01 | -0.112 | 0.051 | -2.191 | 0.028765 | -0.212 | -0.012 |
| **Verbal Memory** |  |  |  |  |  |  |
| **Healthy controls** | **Estimate (Beta)** | **Standard error** | **T value** | ***p-*value** | **95% CI**  **lower bound** | **95% CI**  **upper bound** |
| PGS_SCZ_ P_T_ ≤ 0.01 | 0.024 | 0.040 | 0.616 | 0.538335 | -0.053 | 0.024 |
| **Psychosis** |  |  |  |  |  |  |
| PGS_SCZ_ P_T_ ≤ 0.01 | -0.071 | 0.055 | -1.303 | 0.193157 | -0.178 | -0.071 |
| **Working Memory** |  |  |  |  |  |  |
| **Healthy controls** | **Estimate (Beta)** | **Standard error** | **T value** | ***p-*value** | **95% CI**  **lower bound** | **95% CI**  **upper bound** |
| PGS_SCZ_ P_T_ ≤ 0.01 | -0.030 | 0.037 | -0.808 | 0.419570 | -0.104 | 0.043 |
| **Psychosis** |  |  |  |  |  |  |
| PGS_SCZ_ P_T_ ≤ 0.01 | 0.028 | 0.041 | 0.681 | 0.496252 | -0.053 | 0.028 |

| **Attentional Control** |  | **Table S2.** Cognition polygenic scores (PTs ≤ 5x10^-8^, 1x10^-5^, 0.001, 0.01, 0.05, 0.1 and 0.5) and associations to cognitive domains in healthy controls and individuals with psychosis (Psychosis). |  |  |  |  |
| --- | --- | --- | --- | --- | --- | --- |
| **Healthy controls** | **Estimate (Beta)** | **Standard error** | **T value** | ***p-*value** | **95% CI**  **lower bound** | **95% CI**  **upper bound** |
| PGS_COG_ P_T_ ≤ 5x10^-8^ | 0.023 | 0.029 | 0.776 | 0.438034 | -0.035 | 0.080 |
| PGS_COG_ P_T_ ≤ 1x10^-5^ | 0.023 | 0.029 | 0.800 | 0.423967 | -0.034 | 0.080 |
| PGS_COG_ P_T_ ≤ 0.001 | 0.058 | 0.028 | 2.080 | 0.037854 | 0.003 | 0.112 |
| PGS_COG_ P_T_ ≤ 0.01 | 0.039 | 0.029 | 1.355 | 0.175727 | -0.017 | 0.095 |
| PGS_COG_ P_T_ ≤ 0.05 | 0.020 | 0.029 | 0.694 | 0.487722 | -0.037 | 0.078 |
| PGS_COG_ P_T_ ≤ 0.1 | 0.034 | 0.030 | 1.144 | 0.253132 | -0.025 | 0.093 |
| PGS_COG_ P_T_ ≤ 0.5 | 0.033 | 0.030 | 1.102 | 0.270686 | -0.026 | 0.091 |
| **Psychosis** |  |  |  |  |  |  |
| PGS_COG_ P_T_ ≤ 5x10^-8^ | -0.125 | 0.061 | -2.048 | 0.040967 | -0.245 | -0.005 |
| PGS_COG_ P_T_ ≤ 1x10^-5^ | -0.016 | 0.058 | -0.272 | 0.785474 | -0.130 | 0.098 |
| PGS_COG_ P_T_ ≤ 0.001 | 0.052 | 0.059 | 0.884 | 0.377139 | -0.064 | 0.168 |
| PGS_COG_ P_T_ ≤ 0.01 | 0.105 | 0.059 | 1.784 | 0.074844 | -0.011 | 0.221 |
| PGS_COG_ P_T_ ≤ 0.05 | 0.048 | 0.058 | 0.820 | 0.412564 | -0.067 | 0.162 |
| PGS_COG_ P_T_ ≤ 0.1 | 0.052 | 0.059 | 0.881 | 0.378875 | -0.064 | 0.168 |
| PGS_COG_ P_T_ ≤ 0.5 | 0.014 | 0.059 | 0.232 | 0.816626 | -0.103 | 0.130 |
| **Category Fluency** |  |  |  |  |  |  |
| **Healthy controls** | **Estimate (Beta)** | **Standard error** | **T value** | ***p-*value** | **95% CI**  **lower bound** | **95% CI**  **upper bound** |
| PGS_COG_ P_T_ ≤ 5x10^-8^ | -0.014 | 0.035 | -0.413 | 0.679825 | -0.083 | 0.054 |
| PGS_COG_ P_T_ ≤ 1x10^-5^ | 0.036 | 0.034 | 1.056 | 0.291477 | -0.031 | 0.103 |
| PGS_COG_ P_T_ ≤ 0.001 | 0.030 | 0.033 | 0.917 | 0.359253 | -0.034 | 0.095 |
| PGS_COG_ P_T_ ≤ 0.01 | 0.050 | 0.034 | 1.469 | 0.142187 | -0.017 | 0.116 |
| PGS_COG_ P_T_ ≤ 0.05 | 0.010 | 0.035 | 0.291 | 0.770926 | -0.058 | 0.078 |
| PGS_COG_ P_T_ ≤ 0.1 | 0.020 | 0.035 | 0.577 | 0.564147 | -0.049 | 0.090 |
| PGS_COG_ P_T_ ≤ 0.5 | 0.032 | 0.035 | 0.920 | 0.358009 | -0.037 | 0.101 |
| **Psychosis** |  |  |  |  |  |  |
| PGS_COG_ P_T_ ≤ 5x10^-8^ | -0.081 | 0.046 | -1.772 | 0.076838 | -0.171 | 0.009 |
| PGS_COG_ P_T_ ≤ 1x10^-5^ | -0.057 | 0.044 | -1.289 | 0.197844 | -0.145 | 0.030 |
| PGS_COG_ P_T_ ≤ 0.001 | 0.071 | 0.044 | 1.597 | 0.110798 | -0.016 | 0.158 |
| PGS_COG_ P_T_ ≤ 0.01 | 0.080 | 0.045 | 1.791 | 0.073728 | -0.008 | 0.168 |
| PGS_COG_ P_T_ ≤ 0.05 | 0.039 | 0.044 | 0.885 | 0.376577 | -0.047 | 0.125 |
| PGS_COG_ P_T_ ≤ 0.1 | 0.011 | 0.045 | 0.256 | 0.798390 | -0.077 | 0.100 |
| PGS_COG_ P_T_ ≤ 0.5 | 0.025 | 0.045 | 0.561 | 0.575026 | -0.063 | 0.114 |
| **Composite score** |  |  |  |  |  |  |
| **Healthy controls** | **Estimate (Beta)** | **Standard error** | **T value** | ***p-*value** | **95% CI**  **lower bound** | **95% CI**  **upper bound** |
| PGS_COG_ P_T_ ≤ 5x10^-8^ | 0.016 | 0.022 | 0.734 | 0.463381 | -0.026 | 0.058 |
| PGS_COG_ P_T_ ≤ 1x10^-5^ | 0.044 | 0.021 | 2.072 | 0.038614 | 0.002 | 0.086 |
| PGS_COG_ P_T_ ≤ 0.001 | 0.040 | 0.020 | 1.982 | 0.047835 | 0.000 | 0.081 |
| PGS_COG_ P_T_ ≤ 0.01 | 0.057 | 0.021 | 2.734 | 0.006392 | 0.016 | 0.098 |
| PGS_COG_ P_T_ ≤ 0.05 | 0.036 | 0.021 | 1.677 | 0.093956 | -0.006 | 0.078 |
| PGS_COG_ P_T_ ≤ 0.1 | 0.038 | 0.022 | 1.740 | 0.082307 | -0.005 | 0.081 |
| PGS_COG_ P_T_ ≤ 0.5 | 0.038 | 0.022 | 1.773 | 0.076682 | -0.004 | 0.081 |
| **Psychosis** |  |  |  |  |  |  |
| PGS_COG_ P_T_ ≤ 5x10^-8^ | -0.088 | 0.039 | -2.270 | 0.023607 | -0.165 | -0.012 |
| PGS_COG_ P_T_ ≤ 1x10^-5^ | -0.046 | 0.038 | -1.197 | 0.231729 | -0.121 | 0.029 |
| PGS_COG_ P_T_ ≤ 0.001 | 0.039 | 0.038 | 1.030 | 0.303233 | -0.035 | 0.113 |
| PGS_COG_ P_T_ ≤ 0.01 | 0.055 | 0.038 | 1.435 | 0.151869 | -0.020 | 0.130 |
| PGS_COG_ P_T_ ≤ 0.05 | 0.021 | 0.038 | 0.556 | 0.578773 | -0.054 | 0.097 |
| PGS_COG_ P_T_ ≤ 0.1 | 0.010 | 0.038 | 0.261 | 0.794052 | -0.065 | 0.086 |
| PGS_COG_ P_T_ ≤ 0.5 | 0.008 | 0.038 | 0.198 | 0.843037 | -0.067 | 0.082 |
| **Premorbid IQ NART** |  |  |  |  |  |  |
| **Healthy controls** | **Estimate (Beta)** | **Standard error** | **T value** | ***p-*value** | **95% CI**  **lower bound** | **95% CI**  **upper bound** |
| PGS_COG_ P_T_ ≤ 5x10^-8^ | -0.033 | 0.033 | -0.984 | 0.325633 | -0.098 | 0.033 |
| PGS_COG_ P_T_ ≤ 1x10^-5^ | 0.005 | 0.033 | 0.151 | 0.880343 | -0.060 | 0.070 |
| PGS_COG_ P_T_ ≤ 0.001 | 0.060 | 0.032 | 1.884 | 0.059902 | -0.003 | 0.123 |
| PGS_COG_ P_T_ ≤ 0.01 | 0.078 | 0.032 | 2.418 | 0.015833 | 0.015 | 0.142 |
| PGS_COG_ P_T_ ≤ 0.05 | 0.032 | 0.034 | 0.959 | 0.337649 | -0.034 | 0.098 |
| PGS_COG_ P_T_ ≤ 0.1 | 0.057 | 0.034 | 1.667 | 0.095940 | -0.010 | 0.124 |
| PGS_COG_ P_T_ ≤ 0.5 | 0.084 | 0.034 | 2.497 | 0.012712 | 0.018 | 0.151 |
| **Psychosis** |  |  |  |  |  |  |
| PGS_COG_ P_T_ ≤ 5x10^-8^ | -0.035 | 0.048 | -0.735 | 0.462577 | -0.129 | 0.059 |
| PGS_COG_ P_T_ ≤ 1x10^-5^ | 0.020 | 0.045 | 0.444 | 0.657366 | -0.069 | 0.109 |
| PGS_COG_ P_T_ ≤ 0.001 | 0.057 | 0.046 | 1.246 | 0.213292 | -0.033 | 0.146 |
| PGS_COG_ P_T_ ≤ 0.01 | 0.063 | 0.046 | 1.357 | 0.175351 | -0.028 | 0.154 |
| PGS_COG_ P_T_ ≤ 0.05 | 0.015 | 0.046 | 0.323 | 0.746448 | -0.075 | 0.105 |
| PGS_COG_ P_T_ ≤ 0.1 | 0.021 | 0.046 | 0.442 | 0.658638 | -0.071 | 0.112 |
| PGS_COG_ P_T_ ≤ 0.5 | 0.009 | 0.047 | 0.189 | 0.850535 | -0.083 | 0.101 |
| **Precessing speed** |  |  |  |  |  |  |
| **Healthy controls** | **Estimate (Beta)** | **Standard error** | **T value** | ***p-*value** | **95% CI**  **lower bound** | **95% CI**  **upper bound** |
| PGS_COG_ P_T_ ≤ 5x10^-8^ | -0.011 | 0.027 | -0.398 | 0.690751 | -0.063 | 0.042 |
| PGS_COG_ P_T_ ≤ 1x10^-5^ | 0.030 | 0.026 | 1.155 | 0.248386 | -0.021 | 0.082 |
| PGS_COG_ P_T_ ≤ 0.001 | 0.017 | 0.025 | 0.677 | 0.498427 | -0.032 | 0.067 |
| PGS_COG_ P_T_ ≤ 0.01 | 0.031 | 0.026 | 1.175 | 0.240424 | -0.021 | 0.082 |
| PGS_COG_ P_T_ ≤ 0.05 | 0.025 | 0.027 | 0.944 | 0.345212 | -0.027 | 0.078 |
| PGS_COG_ P_T_ ≤ 0.1 | 0.008 | 0.027 | 0.308 | 0.758437 | -0.045 | 0.062 |
| PGS_COG_ P_T_ ≤ 0.5 | 0.019 | 0.027 | 0.702 | 0.483105 | -0.034 | 0.072 |
| **Psychosis** |  |  |  |  |  |  |
| PGS_COG_ P_T_ ≤ 5x10^-8^ | -0.061 | 0.046 | -1.349 | 0.177692 | -0.151 | 0.028 |
| PGS_COG_ P_T_ ≤ 1x10^-5^ | 0.012 | 0.043 | 0.276 | 0.782728 | -0.073 | 0.097 |
| PGS_COG_ P_T_ ≤ 0.001 | 0.062 | 0.044 | 1.410 | 0.158884 | -0.024 | 0.148 |
| PGS_COG_ P_T_ ≤ 0.01 | 0.081 | 0.044 | 1.832 | 0.067335 | -0.006 | 0.167 |
| PGS_COG_ P_T_ ≤ 0.05 | 0.016 | 0.044 | 0.372 | 0.710359 | -0.069 | 0.102 |
| PGS_COG_ P_T_ ≤ 0.1 | 0.005 | 0.044 | 0.106 | 0.915837 | -0.082 | 0.091 |
| PGS_COG_ P_T_ ≤ 0.5 | -0.020 | 0.044 | -0.462 | 0.644050 | -0.107 | 0.066 |
| **Verbal Learning** |  |  |  |  |  |  |
| **Healthy controls** | **Estimate (Beta)** | **Standard error** | **T value** | ***p-*value** | **95% CI**  **lower bound** | **95% CI**  **upper bound** |
| PGS_COG_ P_T_ ≤ 5x10^-8^ | 0.024 | 0.033 | 0.707 | 0.479534 | -0.042 | 0.089 |
| PGS_COG_ P_T_ ≤ 1x10^-5^ | 0.087 | 0.033 | 2.653 | 0.008115 | 0.023 | 0.152 |
| PGS_COG_ P_T_ ≤ 0.001 | 0.028 | 0.032 | 0.868 | 0.385763 | -0.035 | 0.090 |
| PGS_COG_ P_T_ ≤ 0.01 | 0.061 | 0.033 | 1.856 | 0.063837 | -0.003 | 0.125 |
| PGS_COG_ P_T_ ≤ 0.05 | 0.051 | 0.033 | 1.519 | 0.129156 | -0.015 | 0.116 |
| PGS_COG_ P_T_ ≤ 0.1 | 0.055 | 0.034 | 1.618 | 0.106014 | -0.012 | 0.122 |
| PGS_COG_ P_T_ ≤ 0.5 | 0.060 | 0.034 | 1.763 | 0.078219 | -0.007 | 0.126 |
| **Psychosis** |  |  |  |  |  |  |
| PGS_COG_ P_T_ ≤ 5x10^-8^ | 0.021 | 0.046 | 0.452 | 0.651175 | -0.070 | 0.112 |
| PGS_COG_ P_T_ ≤ 1x10^-5^ | 0.007 | 0.045 | 0.154 | 0.877343 | -0.081 | 0.095 |
| PGS_COG_ P_T_ ≤ 0.001 | -0.002 | 0.045 | -0.050 | 0.960290 | -0.091 | 0.087 |
| PGS_COG_ P_T_ ≤ 0.01 | 0.054 | 0.045 | 1.195 | 0.232640 | -0.035 | 0.143 |
| PGS_COG_ P_T_ ≤ 0.05 | 0.026 | 0.044 | 0.582 | 0.560947 | -0.061 | 0.113 |
| PGS_COG_ P_T_ ≤ 0.1 | 0.012 | 0.045 | 0.272 | 0.785841 | -0.077 | 0.101 |
| PGS_COG_ P_T_ ≤ 0.5 | 0.035 | 0.045 | 0.761 | 0.446662 | -0.055 | 0.124 |
| **Verbal Memory** |  |  |  |  |  |  |
| **Healthy controls** | **Estimate (Beta)** | **Standard error** | **T value** | ***p-*value** | **95% CI**  **lower bound** | **95% CI**  **upper bound** |
| PGS_COG_ P_T_ ≤ 5x10^-8^ | 0.054 | 0.033 | 1.616 | 0.106526 | -0.012 | 0.120 |
| PGS_COG_ P_T_ ≤ 1x10^-5^ | 0.086 | 0.033 | 2.611 | 0.009174 | 0.021 | 0.151 |
| PGS_COG_ P_T_ ≤ 0.001 | 0.024 | 0.032 | 0.752 | 0.452425 | -0.039 | 0.087 |
| PGS_COG_ P_T_ ≤ 0.01 | 0.051 | 0.033 | 1.558 | 0.119573 | -0.013 | 0.116 |
| PGS_COG_ P_T_ ≤ 0.05 | 0.041 | 0.034 | 1.211 | 0.226244 | -0.025 | 0.107 |
| PGS_COG_ P_T_ ≤ 0.1 | 0.036 | 0.034 | 1.045 | 0.296291 | -0.031 | 0.103 |
| PGS_COG_ P_T_ ≤ 0.5 | 0.035 | 0.034 | 1.033 | 0.301691 | -0.032 | 0.102 |
| **Psychosis** |  |  |  |  |  |  |
| PGS_COG_ P_T_ ≤ 5x10^-8^ | -0.063 | 0.050 | -1.245 | 0.213730 | -0.162 | 0.036 |
| PGS_COG_ P_T_ ≤ 1x10^-5^ | -0.028 | 0.048 | -0.579 | 0.562671 | -0.121 | 0.066 |
| PGS_COG_ P_T_ ≤ 0.001 | 0.013 | 0.048 | 0.277 | 0.782020 | -0.082 | 0.109 |
| PGS_COG_ P_T_ ≤ 0.01 | 0.027 | 0.049 | 0.561 | 0.574943 | -0.068 | 0.122 |
| PGS_COG_ P_T_ ≤ 0.05 | 0.021 | 0.048 | 0.444 | 0.656982 | -0.073 | 0.115 |
| PGS_COG_ P_T_ ≤ 0.1 | -0.002 | 0.048 | -0.046 | 0.963055 | -0.097 | 0.093 |
| PGS_COG_ P_T_ ≤ 0.5 | 0.010 | 0.049 | 0.199 | 0.842522 | -0.086 | 0.105 |
| **Working Memory** |  |  |  |  |  |  |
| **Healthy controls** | **Estimate (Beta)** | **Standard error** | **T value** | ***p-*value** | **95% CI**  **lower bound** | **95% CI**  **upper bound** |
| PGS_COG_ P_T_ ≤ 5x10^-8^ | -0,007 | 0,031 | -0,239 | 0,811400 | -0,069 | 0,054 |
| PGS_COG_ P_T_ ≤ 1x10^-5^ | 0,019 | 0,031 | 0,606 | 0,544563 | -0,042 | 0,080 |
| PGS_COG_ P_T_ ≤ 0.001 | 0,079 | 0,030 | 2,647 | 0,008285 | 0,020 | 0,138 |
| PGS_COG_ P_T_ ≤ 0.01 | 0.120 | 0.030 | 3.945 | 0.000086 | 0.060 | 0.179 |
| PGS_COG_ P_T_ ≤ 0.05 | 0.078 | 0.031 | 2.501 | 0.012573 | 0.017 | 0.139 |
| PGS_COG_ P_T_ ≤ 0.1 | 0.079 | 0.032 | 2.469 | 0.013746 | 0.016 | 0.141 |
| PGS_COG_ P_T_ ≤ 0.5 | 0.062 | 0.032 | 1.969 | 0.049230 | 0.000 | 0.124 |
| **Psychosis** |  |  |  |  |  |  |
| PGS_COG_ P_T_ ≤ 5x10^-8^ | -0,064 | 0,037 | -1,708 | 0,088257 | -0,137 | 0,010 |
| PGS_COG_ P_T_ ≤ 1x10^-5^ | -0,044 | 0,036 | -1,207 | 0,227854 | -0,116 | 0,028 |
| PGS_COG_ P_T_ ≤ 0.001 | -0,005 | 0,037 | -0,142 | 0,887198 | -0,077 | 0,067 |
| PGS_COG_ P_T_ ≤ 0.01 | 0.036 | 0.037 | 0.956 | 0.339703 | -0.038 | 0.109 |
| PGS_COG_ P_T_ ≤ 0.05 | 0.021 | 0.037 | 0.563 | 0.573641 | -0.052 | 0.094 |
| PGS_COG_ P_T_ ≤ 0.1 | 0.021 | 0.037 | 0.555 | 0.578827 | -0.052 | 0.093 |
| PGS_COG_ P_T_ ≤ 0.5 | 0.017 | 0.037 | 0.473 | 0.636570 | -0.055 | 0.090 |

**Table S3.** Pearson Correlations between symptoms/substance abuse and cognitive phenotypes

|  | | Working Memory | Cognitive Composite | Premorbid IQ NART |
| --- | --- | --- | --- | --- |
| PANSS  Wallwork Positive | Correlation | -.102^*^ | -.202^**^ | -.177^**^ |
|  | *p*-value | .016 | .000 | .000 |
|  | N | 557 | 526 | 629 |
| PANSS  Wallwork Negative | Correlation | -.102^*^ | -.195^**^ | -.120^**^ |
|  | *p*-value | .016 | .000 | .003 |
|  | N | 556 | 525 | 628 |
| PANSS  Wallwork Disorganized | Correlation | -.195^**^ | -.277^**^ | -.311^**^ |
|  | *p*-value | .000 | .000 | .000 |
|  | N | 556 | 525 | 628 |
| PANSS  Wallwork Excited | Correlation | -.041 | -.027 | -.136^**^ |
|  | *p*-value | .338 | .538 | .001 |
|  | N | 556 | 525 | 628 |
| PANSS  Wallwork Depressed | Correlation | -.035 | -.033 | .018 |
|  | *p*-value | .410 | .454 | .646 |
|  | N | 558 | 527 | 630 |
| AUDIT | Correlation | .055 | .070 | -.043 |
|  | *p*-value | .270 | .162 | .379 |
|  | N | 407 | 402 | 419 |
| DUDIT | Correlation | -.004 | .000 | -.095^*^ |
|  | *p*-value | .932 | .993 | .045 |
|  | N | 423 | 418 | 446 |
| Duration of illness | Correlation | -.089* | -0.013 | .139** |
|  | *p*-value | 0.039 | 0.86 | 0.001 |
|  | N | 541 | 184 | 614 |

*=sig. at *p* > .05; **= sig. at *p* > .01
AUDIT: Alcohol Use Disorder Test
DUDIT: Drug Use Disorder Test
PANSS: PANSS: Positive and Negative Syndrome in Schizophrenia
Walwork = Wallwork five-factor component derived from the Positive and Negative Syndrome scale.

**Table S4.** Example output showing the regression for Working Memory and association to PGS_COG_ P_T_ ≤ 0.01 after correcting for age, gender, genotyping batch, ancestry components, symptoms, substance use and duration of illness.

|  | Unstandardized Coefficients | | Standardized Coefficients | t | *p*-value | 95.0% CI for Beta | |
| --- | --- | --- | --- | --- | --- | --- | --- |
| (Constant) | jan.96 | 6.152 |  | 0.319 | 0.75 | -10.156 | 14.076 |
| Gender | -0.042 | 0.111 | -0.024 | -0.38 | 0.704 | -0.261 | 0.176 |
| Age | -0.016 | 0.006 | -0.187 | -2.654 | 0.008 | -0.027 | -0.004 |
| Genotyping batch #1 | -0.207 | 0.31 | -0.041 | -0.667 | 0.506 | -0.817 | 0.404 |
| Genotyping batch #2 | 0.345 | 0.158 | 0.136 | feb.19 | 0.029 | 0.035 | 0.656 |
| Genotyping batch #3 | 0.136 | 0.131 | 0.066 | 1.035 | 0.302 | -0.123 | 0.394 |
| Genotyping batch #4 | 0.112 | 0.444 | 0.015 | 0.252 | 0.801 | -0.763 | 0.987 |
| Genotyping batch #5 | -0.369 | 0.341 | -0.065 | -1.082 | 0.28 | -1.041 | 0.303 |
| Prinicipal component #01 | -20.303 | 41.053 | -0.057 | -0.495 | 0.621 | -101.149 | 60.542 |
| Prinicipal component #02 | 6.441 | 15.045 | 0.041 | 0.428 | 0.669 | -23.187 | 36.069 |
| Prinicipal component #03 | 0.037 | 13.071 | 0 | 0.003 | 0.998 | -25.704 | 25.778 |
| Prinicipal component #04 | 5.855 | 13.913 | 0.037 | 0.421 | 0.674 | -21.543 | 33.254 |
| Prinicipal component #05 | 9.279 | 8.346 | 0.083 | 1.112 | 0.267 | -7.156 | 25.714 |
| Prinicipal component #06 | -8.726 | 12.254 | -0.05 | -0.712 | 0.477 | -32.858 | 15.406 |
| Prinicipal component #10 | -5.011 | 6.881 | -0.045 | -0.728 | 0.467 | -18.561 | aug.54 |
| Prinicipal component #12 | -12.649 | 7.427 | -0.104 | -1.703 | 0.09 | -27.275 | 1.977 |
| Prinicipal component #15 | -2.956 | 6.364 | -0.028 | -0.464 | 0.643 | -15.489 | 9.578 |
| AUDIT* score | 0.008 | 0.009 | 0.068 | 0.976 | 0.33 | -0.009 | 0.025 |
| DUDIT* score | -5.37E-05 | 0.008 | 0 | -0.006 | 0.995 | -0.017 | 0.017 |
| PANSS* positive | -0.011 | 0.015 | -0.053 | -0.73 | 0.466 | -0.042 | 0.019 |
| PANSS negative | -0.011 | 0.011 | -0.068 | -1.009 | 0.314 | -0.032 | 0.01 |
| PANSS disorganized | -0.084 | 0.026 | -0.237 | -3.218 | 0.001 | -0.136 | -0.033 |
| PANSS exitative | 0.022 | 0.03 | 0.051 | 0.756 | 0.451 | -0.036 | 0.081 |
| PANSS depressive | 0 | 0.019 | 0.002 | 0.025 | 0.98 | -0.037 | 0.038 |
| Duration of illness | -0.007 | 0.01 | -0.046 | -0.671 | 0.503 | -0.026 | 0.013 |
| PGS_COG_ at PT ≤ .01 | -0.142 | 0.525 | -0.016 | -0.271 | 0.787 | -1.176 | 0.892 |

AUDIT: Alcohol Use Disorder Test
DUDIT: Drug Use Disorder Test
PANSS: Positive and Negative Syndrome in Schizophrenia. Walwork five-factor component.

**Figure S1.** Associations between PGS_COG_ and cognitive phenotypes for both individuals with psychosis and healthy controls.

| 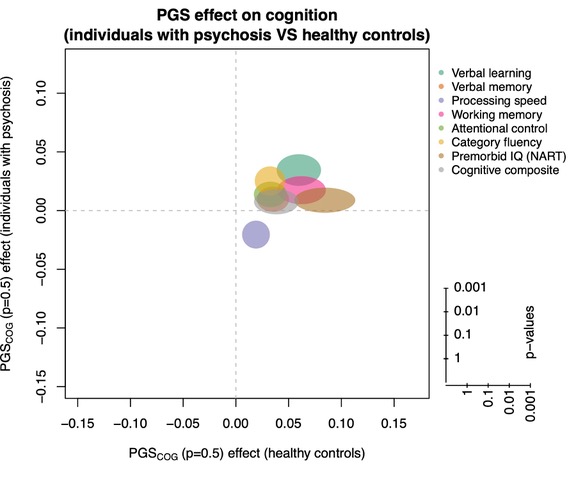 | 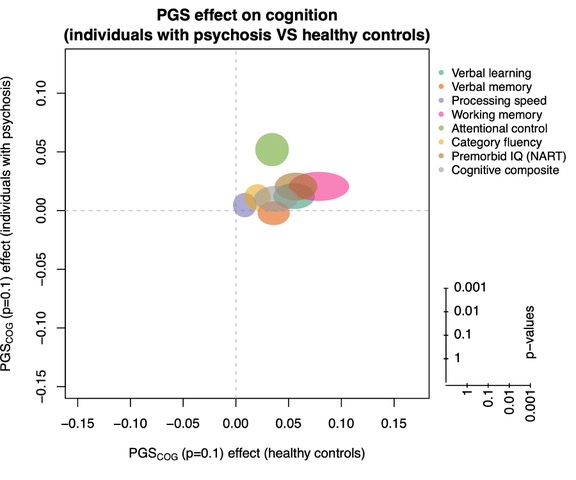 |
| --- | --- |
| 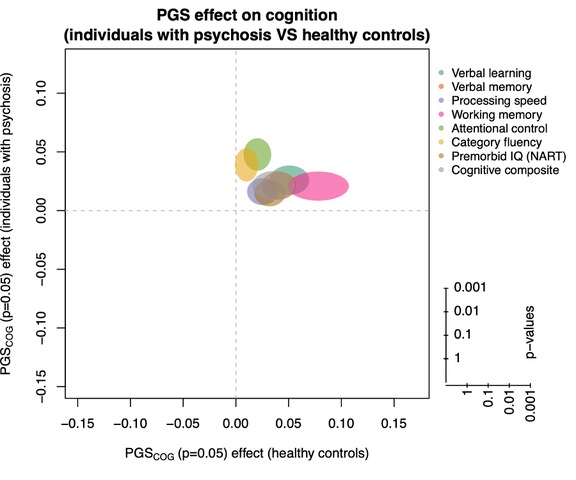 | 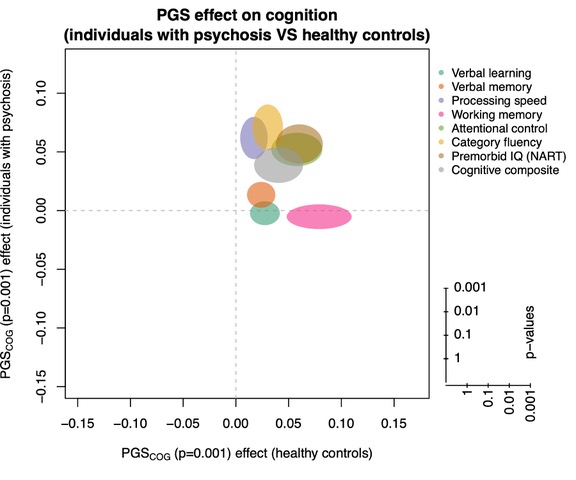 |
| 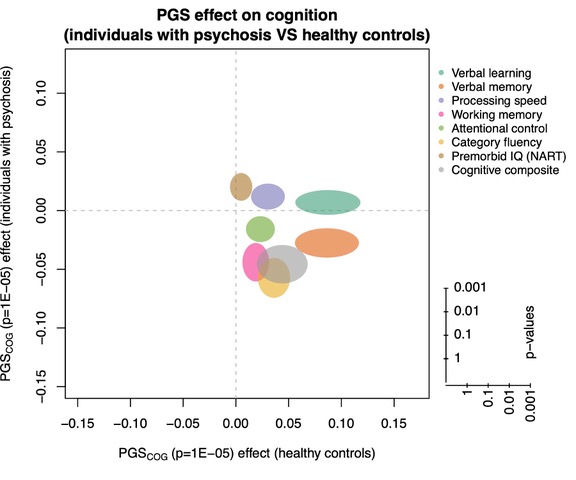 | 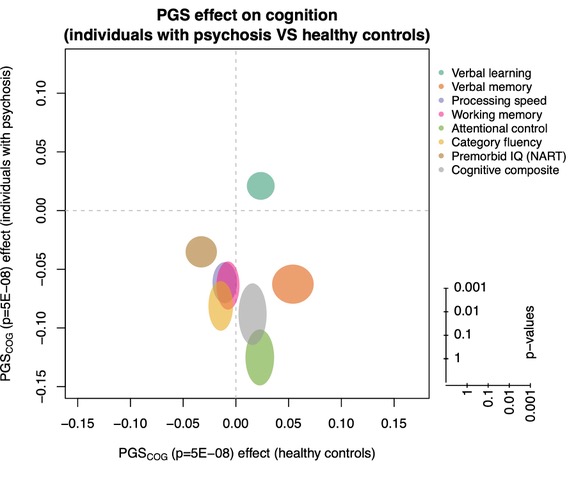 |

The axes denote the size of the Betas in the regressions. The size of the bubbles express the *p*-value of the associations, with larger bubbles indicating progressively smaller *p*-values.
